# Laparoscopic vs. Transabdominal Treatment for Overflow Fecal Incontinence Due to Residual Aganglionosis or Transition Zone Pathology in Hirschsprung's Disease Reoperation

**DOI:** 10.3389/fped.2021.600316

**Published:** 2021-04-27

**Authors:** Feng Chen, Xiaoyu Wei, Xiaohua Chen, Lei Xiang, Jiexiong Feng

**Affiliations:** ^1^Department of Pediatric Surgery, Fujian Medical University Union Hospital, Fuzhou, China; ^2^Department of Pediatric Surgery, Tongji Hospital, Tongji Medical College, Huazhong University of Science and Technology, Wuhan, China

**Keywords:** Hirschsprung disease, laparoscopy, reoperative surgery, incontinence, outcomes

## Abstract

**Objective:** The aim of this study was to describe the details of laparoscopic-assisted reoperative surgery for Hirschsprung's disease (HSCR) with overflow fecal incontinence, and to retrospectively compare laparoscopic-assisted surgery with transabdominal pull-through surgery.

**Methods:** We retrospectively analyzed patients with HSCR with overflow fecal incontinence after the initial surgery in our center between January 2002 and December 2018. Pre-operative, peri-operative, and post-operative data were recorded for statistical analysis.

**Results:** Thirty patients with overflow fecal incontinence after initial megacolon surgery [17 who underwent transanal pull-through (TA-PT) and 13 who underwent laparoscopic-assisted pull-through (LA-PT)] required a secondary surgery [reoperation with LA-PT (LAR-PT) (*n* = 16) or reoperation with transabdominal pull-through (TR-PT) (*n* = 14)]. Indications for reoperation were residual aganglionosis (RA) (7/30, 23.3%) or transition zone pathology (TZP) (23/17, 76.7%). Blood loss was significantly decreased in the LAR-PT group (75 ± 29.2 ml) compared to the TR-PT group (190 ± 51.4 ml) (*P* = 0.001). The length of hospital stay was significantly shorter in the LAR-PT group (10 ± 1.5 days) than that in the TR-PT group (13 ± 2.4 days). No significant differences were found between two groups in surgical methods, defecation function score, or post-operative complications except for wound infection (LAR-PT vs. TR-PT 0 vs. 28.6%, *P* < 0.05).

**Conclusions:** It is necessary to make a comprehensive analysis of the causes of fecal incontinence after HSCR surgery and make an accurate judgment using appropriate methods. If a reoperation was inevitable for patients with overflow fecal incontinence due to RA or TZP, a comprehensive evaluation prior to the operation is required to maximize the benefit from reoperation. Although laparoscopic reoperation with heart-shaped anastomosis was safe and feasible for patients with failed initial Soave technique, unnecessary reoperation should be avoided as much as possible.

## Introduction

Since Swenson (1) first successfully performed surgical treatment for Hirschsprung's disease (HSCR) in 1948, many modifications and advanced techniques have been used to improve this intervention. With the development of the less invasive surgical techniques, quality of life for patients with HSCR have been improved vastly. However, many post-operative complications may occur, such as constipation, abdominal distention, soiling, and incontinence (defined as a patient with involuntary bowel movements) (2). Fecal continence refers to the ability to voluntary defecate without soiling (defined as a patient with voluntary bowel movements, wherein the patient had more than one involuntary bowel movement between two voluntary bowel movements with few or only liquid feces) and using an enema. The physiologic elements needed to maintain continence include intact anal sensation, voluntary sphincter control, and appropriate colonic motility (3). Once these constituent become damaged, partial or total fecal incontinence will occur. This type of fecal incontinence is defined as true incontinence.

In theory, children with HSCR have an anatomically intact continence mechanism after birth, and all HSCR radical surgical techniques are designed to preserve intact anal sensation and voluntary sphincter control mechanism. Therefore, children with HSCR after surgery should not have true fecal incontinence. However, post-operative fecal incontinence may still occur for the following two reasons. Firstly, the intact anal sensation and the sphincter accidentally injured during surgery may lead to the occurrence of true incontinence. Secondly, the obstruction of the distal colon is not completely relieved during the operation, which led to the persistence of post-operative distal colon obstruction. The causes of the post-operative obstruction could be anatomical or histopathological: including residual aganglionosis (RA), transitional zone pathology (TZP), stricture, a retained dilated segment, or a Soave muscular cuff (4, 5). In mechanical bowel obstruction, a blockage in the distal colon causes the pressure in the proximal colon to increase, and over time, the rectum and anal sphincters expand and relax. When the colon pressure gradually exceeds the anal pressure, feces overflow from the anus, which is called overflow fecal incontinence.

Currently, most patients and surgeons have accepted the concept of minimally invasive laparoscopic surgery, which has become the most popular surgical procedure for HSCR (6–8). The aim of this study was to share our experiences in the treatment of overflow fecal incontinence due to TZP or RA, with comparing the endpoints of open surgery and laparoscopic surgery for HSCR.

## Methods

### Materials

**Figure 1:**
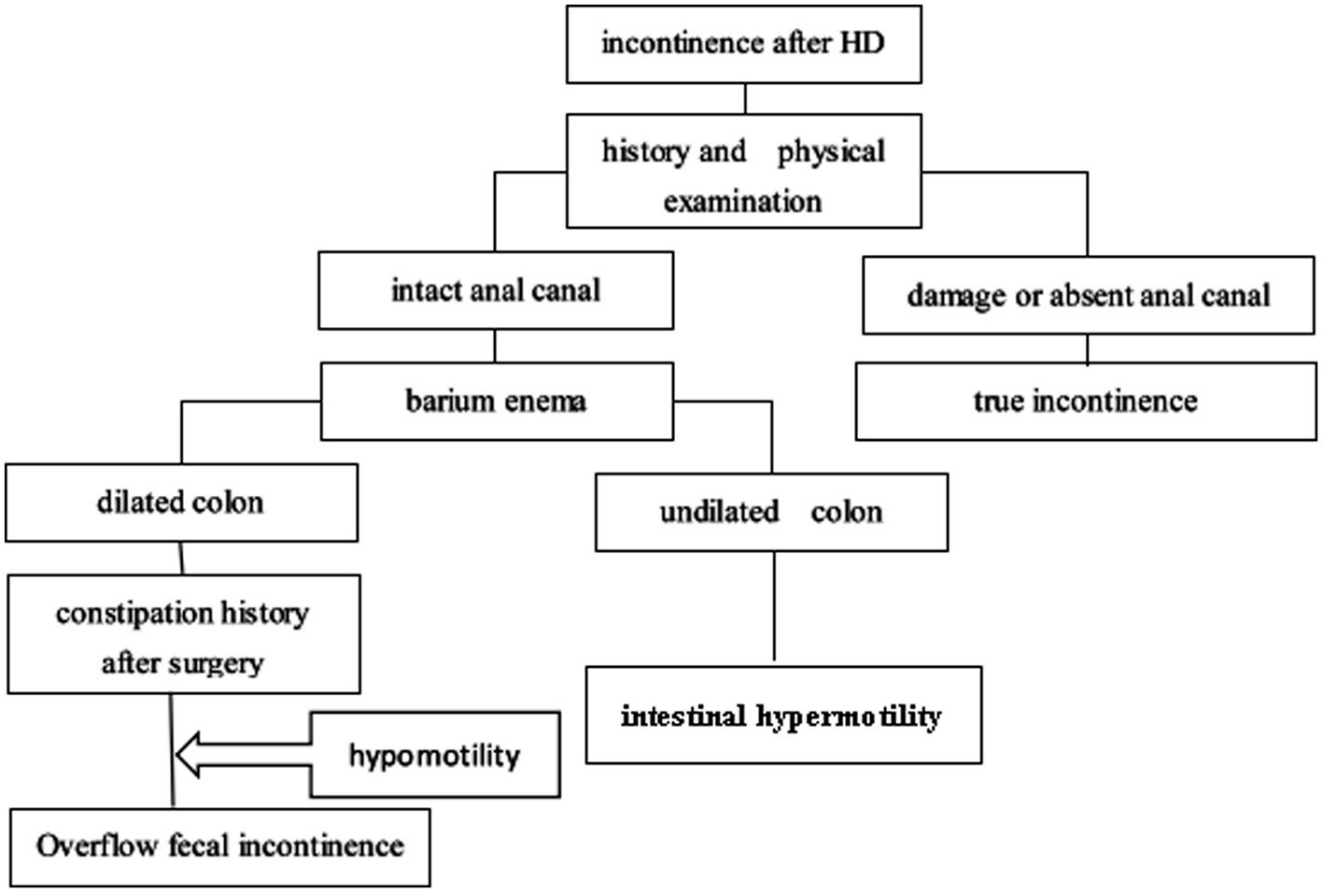
Incontinence diagnosis procedure.

We retrospectively analyzed all patients with HSCR in our center between January 2002 and December 2018. This study was approved by the Institutional Review Board of Tongji Medical College. Patients were included if they continually complained of soiling or fecal incontinence for more than 12 months after the initial surgery. Patients were excluded if they were younger than 4 years (before the age of toilet training), had total colonic aganglionosis, had hypothyroidism, had neurological diseases, or had a colostomy at the time of evaluation. At last, 71 patients were subjected to our protocol. Signed consent form, medical history, physical examination (checking the integrity of the anal canal), and barium enema (BE) were obtained from each subject. If patients had an intact anal canal, they had the potential for bowel control, and may become continent over time. It was vital to carefully determine whether the anus was intact. We classified the type of fecal incontinence in children with incontinence after HSCR surgery according to an algorithm (Figure 1). (1) The anal canal was intact: if a BE showed that the colon was dilated, and the patient had a history of constipation, then the disorder was considered overflow fecal incontinence caused by intestinal hypomotility; if a BE showed an undiluted colon, it was considered as intestinal hypermotility, and loperamide, pectin, and dietary modifications were offered. (2) The anal canal was damaged in a previous surgery: this condition was considered as true incontinence, and daily colonic irrigation was chosen to improve the patient's quality of life by reducing the frequency of soiling.

According to the above scheme and process, 47 patients with overflow fecal incontinence were evaluated. After the primary operation in other hospital, all children experienced constipation, soiling or fecal incontinence gradually, which eventually attracted the attention of their parents. Although intermittent anal dilation, glycerine or colonic irrigation were given during the course of the disease, the symptoms persisted. We chose conservative treatment, including diet control, laxative treatment, anal dilation, enema, and colonic irrigation for at least 3 months. A secondary surgery was considered after such conservative treatments failed. Among them, 12 patients were successfully treated with conservative treatment, and 35 patients underwent reoperation. Among these 35 patients, five cases were excluded to minimize bias because these patients initially underwent laparotomy. We ultimately selected 30 patients with HSCR with overflow fecal incontinence as subjects. All children underwent the primary operation and treatment in other hospitals. Furthermore, the type of primary operation was either transanal or laparoscopic-assisted pull-through (TA-PT or LA-PT, respectively) and all patient's anastomosis was performed in Soave technique.

Before reoperation, surgeons reviewed the patients' medical history and performed physical examinations, BE, anorectal manometry, and histochemical acetylcholinesterase (AChE) staining of the rectal biopsy. All patients underwent rectal biopsy of mucosal/submucosal or full thickness, which was performed 3 and 6 cm above the dentate line. The interpretation of AChE staining was similar to that described by El-Badawi (9). All procedures were performed by a very experienced and single pediatric surgeon at the department of Pediatric Surgery of Tongji Hospital, Huazhong University of Science and Technology (Wuhan, China). Before deciding to proceed with the operation, the children's guardians were properly informed of all risks and potential benefits, and surgical procedure decision was made according to individual circumstances and preferences of each family. The diagnosis was confirmed by intraoperative frozen section, and furtherly confirmed by the post-operative pathology. The speculative extent of the diseased intestine was determined by the surgeon prior to the operation, based on the BE and a 24-h delayed abdominal radiograph (24hDAR, barium residue). During the operation, the length of the resected bowel was determined by intraoperative frozen section and pre-operative findings. Furthermore, an experienced pathologist was assigned to examine the submucosal ganglia and nerve trunk in the intestine. Aganglionosis was defined as the absence of ganglion cells in the submucosa and myenteric plexus with hypertrophied and hyperplastic nerves. A transition zone was defined as the presence of ganglion cells in the submucosa or myenteric plexus with hypertrophied and hyperplastic nerves.

The pre-operative, intraoperative, and post-operative data of the children were recorded in detail in both groups, including demographic information, operation time, type of primary PT, type of repeat PT, length of the aganglionic segment, early complications of surgery and short-term outcome [anastomotic leak, twisted PT, wound infection, perforation, Hirschsprung-associated enterocolitis (HAEC), soiling, and uroschesis], and late complications and long-term outcome (constipation, stricture, fistula, soiling, and HAEC).

### Surgical Procedures

#### The Laparoscopic-Assisted Pull-Through Reoperative Technique With Heart-Shaped Anastomosis

A 1-cm skin incision was first made below the umbilical margin, and a 5-mm trocar was placed into the abdomen after incising the peritoneum. Two 5-mm trocars were then placed on both sides of the umbilicus. Next, we explored the abdomen by inserting a 5-mm, 30° optic fiber, found the suspected intestinal lesion, and cut 2–3 pieces for colonic seromuscular level biopsies for rapid frozen pathological examination to determine whether normal ganglion cells were present in the submucosal nerve plexus. Then, according to the pre-operative examination, intraoperative exploration, and biopsy results, we determined the resection range. Next, the adhesions were gently and completely separated, and the affected portions of the descending, transverse and ascending colon were then mobilized distally beyond the peritoneal reflection level of the rectum and 5 cm proximal to the most distal biopsy site showing normal ganglion cells by cutting down the mesentery using an ultrasonic scalpel. Finally, a heart-shaped anastomosis was performed: the sponge forceps were inserted into the upper part of rectum through anus. With the assistance of laparoscopy, a segment of colon which had been mobilized was clamped. Then the colon was pulled out and everted. After the distal colon was incised, the affected intestine was pulled out until the normal colon was determined by intraoperative biopsy. Subsequently, the affected intestine was removed, and a longitudinal incision was made at the back wall of anorectal tube, about 0.5–1.0 cm above the dentate line to avoid damage to the sphincter and anal sinus. Finally, the remaining rectum and the normal colon were sutured in layers. The anastomosis was high at the front and low at the back, and the front was about 3–4 cm higher than the back, similar to the heart shape [The new method was reported by our center (10, 11)].

#### The Transabdominal Pull-Through Reoperative Procedure With Heart-Shaped Anastomosis

An incision was made in the vertical paramedian and left lower abdomen. The following abdominal and anal procedures were similar to the laparoscopic surgery described above.

After surgery, all patients had an anal tube placed for 7–14 days to facilitate defecation and to prevent anastomotic rupture. The first rectal digital examination was performed after 14 days, and routine anal dilatation was required in all patients once or twice weekly for at least 3 months.

### Statistical Analysis

Statistical analyses for all variables were performed using SPSS Version 20.0. The normality was checked for each parameter using Shapiro-Wilk test. The results are expressed as ranges and means ± standard deviations (SD). Fisher's exact test was used to analyze dichotomous variables, and Student's *t*-test was used to analyze continuous parameters. The Kruskal-Wallis test was used to analyze the defecation function scores. A *P*-value <0.05 was considered statistically significant.

## Results

### Distribution of Patient Data

All 30 children underwent the first surgical treatment for HSCR in other hospitals, including 17 patients who underwent TA-PT and 13 who underwent LA-PT. Review of the surgical records and post-operative routine pathology showed that the rectum or rectosigmoid had been resected, and the normal intestine with normal ganglions was anastomosed at the distal end. The lengths of removed segments ranged from 12 to 25 cm. Details of the patient characteristics are listed in Supplementary Table 1, including symptoms, primary operation, pre-operative examination, reoperation, and histopathological findings. Among all patients, BE showed HSCR, and 24hDAR after BE revealed that barium remaining in the transverse colon, sigmoid colon or the proximal to descending colon. The secondary operation consisted of LAR-PT (*n* = 16) or TR-PT (*n* = 14). For all patients, the histopathology findings showed RA (7 of 30, 23.3%) or TZP (23 of 30, 76.7%) after reoperation.

### Information and Surgical Features of Patients

Table 1 shows that there were no differences in gender, age, follow-up time, or length of bowel resection between the groups. However, significant differences were observed in the volume of blood loss and days spent in the hospital. Blood loss in the LAR-PT group (75 ± 29.2 ml) was significantly less than that in the TR-PT group (190 ± 51.4 ml). The length of hospital stay in the LAR-PT group (10 ± 1.5 days) was significantly shorter than TR-PT group (13 ± 2.4 days).

**Table 1:** Information and surgical features of patients.

	**LAR-PT (*n* = 16)**	**TR-PT (*n* = 14)**	***P*-value**
	**Mean ± SD**	**Mean ± SD**	
Male:Female^a^	8:1	7:1	0.735
Age Redo (month)^a^	60.44 ± 13.24	58.93 ± 14.08	0.765
Follow-up time (month)^a^	33 ± 11.5	30 ± 15.6	0.656
Blood loss (ml)^b^	75 ± 29.2	190 ± 51.4	0.001
Length of resection (cm)^a^	51 ± 16	49 ± 8.2	0.722
Length of hospital stay (day)^b^	10 ± 1.5	13 ± 2.4	0.008

*LAR-PT, reoperation by the laparoscopic-assissted pull through; TR- PT, reoperation with the transabdominal pull through*.

a *P > 0.05*.

b *P < 0.05*.

The distribution of surgical procedures was not significantly different between children underwent primary and repeated surgery, as shown in Supplementary Table 2 (*P* > 0.05). Supplementary Table 3 shows the Heikkinen (12) clinical continence scoring criteria, which divided the defecation continence into four levels: excellent, good, fair, and poor. All patients were followed up by telephone or face-to-face meeting after surgery for more than 1 year to obtain the corresponding defecation function scores for the LAR-PT and TR-PT groups, as shown in Supplementary Table 4. However, no significant difference was observed in the defecation continence score between two groups. The results shown in Supplementary Tables 2, 4 suggest that no significant correlation exists between the choice of surgical approach and post-operative defecation continence function.

### Post-operative Complications and Outcome

No significant differences in post-operative early and late complications were found between the two groups during the follow-up period, except for wound infection. We defined early post-operative complications and short-term outcome (listed in Table 2) as those that occurred within 1 year, and late post-operative complications and long-term outcome (listed in Table 3) as those that remained 1 year post reoperation. Among the early complications, there were four cases (28.6%) of incision infection and two case (14.3%) of urinary retention in the TR-PT group; none of the patients in the LAR-PT group experienced these conditions. The children with incision infections were treated by cleansing and dressing the incision, and the incision then healed after debridement. Children with urinary retention were treated by placing an indwelling catheter for 1 week to restore urinary function. HAEC and soiling occurred in the two groups, during both the early and late complication stages, but no other complications such as constipation or incontinence occurred. The symptoms of HAEC improved with anti-inflammatory and colonic treatments, colon lavage, and regulation of the intestinal microflora. Among the early and late complications, the incidence rates of HAEC decreased from 43.8 to 25% in the LAR-PT group and from 50 to 21.4% in the TR-PT group, respectively. Similarly, the symptoms of soiling were improved with the training of defecation function and the relief of anal sphincter spasms. The incidence of soiling decreased from 43.8 to 12.5% in the LAR-PT group and from 50 to 14.3% in the TR-PT group. However, no significant difference was observed in the incidence of HAEC or soiling between the two groups when comparing both early and late complications (all *P* > 0.05).

**Table 2:** Early post-operative complications and short-term outcome.

	**LAR-PT (*n* = 16)**	**TR-PT (*n* = 14)**	***P*-value**
Anastomotic leak, *n* (%)	0 (0)	0 (0)	NS
Twisted PT, *n* (%)	0 (0)	0 (0)	NS
Wound infection, *n* (%)^b^	0 (0)	4 (28.6%)	0.037
Perforation, *n* (%)	0 (0)	0 (0)	NS
Enterocolitis, *n* (%)^a^	7 (43.8%)	7 (50%)	0.509
Constipation, *n* (%)	0 (0)	0 (0)	NS
Soiling, *n* (%)^a^	7 (43.8%)	7 (50%)	0.509
Incontinence, *n* (%)	0 (0)	0 (0)	NS
Uroschesis, *n* (%)^a^	0 (0)	2 (14.3%)	0.209

*LAR-PT, reoperation by the laparoscopic-assissted pull through; TR- PT, reoperation with the transabdominal pull through; NS, not significant*.

a *P > 0.05*.

b *P < 0.05*.

**Table 3:** Late post-operative complications and long-term outcome.

	**LAR-PT (*n* = 16)**	**TR-PT (*n* = 14)**	***P*-value**
Constipation, *n* (%)	0 (0)	0 (0)	NS
Stricture, *n* (%)	0 (0)	0 (0)	NS
Fistula, *n* (%)	0 (0)	0 (0)	NS
Enterocolitis, *n* (%)^a^	4 (25%)	3 (21.4%)	0.581
Soiling, *n* (%)^a^	2 (12.5%)	2 (14.3%)	0.648
Incontinence, *n* (%)	0(0)	0 (0)	NS

*LAR-PT, reoperation by the laparoscopic-assissted pull through; TR- PT, reoperation with the transabdominal pull through; NS, not significant*.

a *P > 0.05*.

Long-term follow-up was obtained in both groups after operation, and there was no difference in follow-up time (Listed in Table 1). During the first month of follow-up after surgery, most patients had more than 15 bowel movements per day frequently; however, the frequency of bowel movements decreased significantly over time. At the last follow-up, 26 patients had complete fecal control (the frequency of stools was 2–3 times a day), and only 4 patients had soiling occasionally without social issues.

## Discussion

Children with megacolon can benefit from surgical treatment. However, even after a successful surgery, some patients still experience a wide array of stooling disorders. These disorders can range from intermittent enterocolitis to far more significant issues, such as severe stool retention and intestinal obstruction, as well as incontinence (13, 14). In 2010, Levitt et al. (15) reported a need for a high index of suspicion for those patients who were not recovering well after a PT procedure to detect those with anatomically or histopathologically correctable problems. Although there are many reasons for distal colon obstruction after HSCR surgery, most current reports focus on RA or TZP (16). In our study, 30 patients were eventually confirmed to have RA or TZP despite undergoing TA-PT or LA-PT surgery. Children with obstruction of the distal colon might gradually develop constipation, soiling, or fecal incontinence.

Based on epidemiological research, Rajindrajith et al. (17) reported that the incidence of fecal incontinence in 2,686 children was 2%. They also discovered that fecal incontinence contributed to developing an unsociable personality, social dysfunction, and mental disorders in children. In turn, mental and psychological disorders could aggravate incontinence. The appearance of constipation after HSCR surgery is often mild and lagging. The patient's parents may not notice the symptoms or may manage the symptoms inappropriately, which results in prolonged problems and eventually leads to “fecal incontinence.” Through our diagnostic process, we can identify the type of fecal incontinence. Patients with overflow fecal incontinence have bloating stools in their underwear, and a massive fecal mass can be reached by digital rectal examination. Anorectal manometry can help to understand the peristaltic function of the intestine by detecting the rectoanal inhibitory reflex (RAIR), the anal canal resting pressure, the rectal resting pressure, and the anal canal peristalsis frequency, rhythm, amplitude, and compliance. In the current study, increased anorectal pressure was detected in all of the patients, and a RAIR was induced in most children. The BE could show the shape of the intestine, which appeared as a narrow or dilated segment. Previous studies (18) in our center showed the density of interstitial cells of Cajal was significantly decreased in the bowel segments that displayed barium retention. The 24hDAR reflected the peristalsis and transport of the intestine and might be a valuable tool for predicting the length of bowel resection in patients with HSCR. As a result, a preliminary determination could be made on the range of the lesion in the colon and the length of the pre-operatively resected bowel (19). Of course, the frozen section pathology results during surgery could be referenced to determine the length of the intestine to be removed. In all 30 cases, pre-operative 24hDAR showed barium residue in the distal end of the ascending colon or in the transverse colon, and intraoperative frozen section suggested that normal ganglion cells did not exist before the transverse colon to the distal end of the ascending colon. Therefore, the pre-operative 24hDAR might be used as a good reference for the extent of suspicious intestinal biopsy in surgery, and ultimately for the achievement of the goal that removing all diseased bowel. Finally, all patients underwent left hemicolectomy or subtotal colectomy based on the results of intraoperative frozen pathology and pre-operative findings. In addition, the results of the routine pathological examination of the resected bowel segment were consistent with our pre-operative judgment and intraoperative frozen pathology. No children had recurrence of constipation and fecal incontinence through long-term follow-up.

RA or TZP will cause the post-operative symptoms of persistent constipation, eventually leading to the occurrence of soiling or fecal incontinence, thus, a strong indication exists to remove the diseased intestine. However, the risk of a repeated surgery is greater than the primary surgery. When the primary operation was laparoscopic-assisted or transanal surgery, the intraoperative abdominal cavity was less disturbed. As a result, the adhesions seen during the reoperation were relatively mild, as noted by many authors (20, 21), allowing greater feasibility of laparoscopy for the secondary surgery. In our study, none of the children undergoing laparoscopic surgery were converted to open surgery. Recent studies have shown that LA-PT could significantly reduce the surgical trauma for children, speed up the recovery time of bowel movement, promote early recovery, reduce hospitalization time, and result in more visually pleasing incision scars (22, 23). Our results were similar. Compared with laparoscopic surgery, open surgery had more trauma, more bleeding and greater impact on intestinal function, leading to a longer recovery time, slower recovery of intestinal peristalsis, and a longer duration of hospitalization. However, regarding the secondary surgery in our study, no significant differences were found in the incidence of post-operative constipation and HAEC complications between the two groups. Langer (22) and Weber et al. (24) also reported no correlation in the outcomes based on the type of PT, either at primary PT or repeated PT. Of course, when deciding the surgical technique, the preferences of the patient's guardians and the technical level of the surgeons should be considered. Open surgery might be a better choice in some circumstances, such as when a child presented with severe abdominal adhesions or a frozen pelvic cavity pre-operatively. With the improvement in laparoscopic techniques and the accumulation of surgical experience in this field, laparoscopic surgery for HSCR reoperation may become more popular over time.

Until now, there is no recommendation for the method of anastomosis during HSCR reoperation. Schweizer et al. (25) believed that the Duhamel procedure was the best method in complicated cases because this procedure considers the anatomy of the pelvis and has advantages in various surgical techniques, including surgical procedure caused alterations in the pelvis. Sheng (26) and Teitelbaum (27) recommended the Soave procedure as the first choice for secondary anastomosis. However, in our cases, the initial anastomosis was performed in Soave technique, so we chose the heart-shaped anastomosis for patients in whom the mucosectomy was difficult to perform. In addition, the “heart-shaped anastomosis” with high front and low back was performed to expand the anastomotic caliber so as to effectively avoid post-operative stenosis. Since 2000's, the modified operation for HSCR had been used in many medical centers in China. As for the long-term outcomes (11), the incidence of anal sphincter dysfunction and intraoperative nerve injury, as well as the incidence of post-operative constipation and soiling have been reduced.

Our research showed no significant difference in the stooling, regardless of the type of PT used for primary PT or repeated PT. This result was consistent with those reported by other scholars (25, 28, 29), indicating that the management of recurrent HSCR requires a detailed and cogent evaluation of the patient's condition by an experienced team of pediatric surgeons and pathologists. Surgical and other treatment options should be elaborated and personalized, rather than focusing solely on the choice of surgical technique.

The limitations of our study are its retrospective nature and small sample size. Larger prospective randomized studies and detailed long-term follow-ups are required to explore additional differences between these two procedures.

## Conclusion

It is necessary to make a comprehensive analysis of the causes of fecal incontinence after HSCR surgery and make an accurate judgment with various appropriate methods. Children with overflow fecal incontinence after PT for HSCR may have RA or TZP of the bowel and may benefit from re-operative treatment. When a reoperation was inevitable in a patient with overflow fecal incontinence after initial HSCR surgery, it was very important to select the appropriate operation. Laparoscopically assisted reoperation with heart-shaped anastomosis was feasible and more advantageous than open surgery, with less operative bleeding, faster recovery, and shorter hospital stay. Of course, larger prospective randomized studies and detailed long-term follow-ups are needed. Even if precise reoperation and other comprehensive treatments may cure RA or TZP, we should reduce the necessity of reoperation as least as possible.

## Data Availability Statement

The original contributions presented in the study are included in the article/Supplementary Material, further inquiries can be directed to the corresponding author/s.

## Ethics Statement

The studies involving human participants were reviewed and approved by the Institutional Review Board of Tongji Medical College. Written informed consent to participate in this study was provided by the participants' legal guardian/next of kin.

## Author Contributions

FC and JF: study conception and design. FC and LX: data acquisition. FC, XW, LX, and XC: analysis and data interpretation. FC: drafting of the manuscript. XW and JF: critical revision. All authors contributed to the article and approved the submitted version.

## Conflict of Interest

The authors declare that the research was conducted in the absence of any commercial or financial relationships that could be construed as a potential conflict of interest.

## Abbreviations

HSCR: Hirschsprung's disease
RA: residual aganglionosis
TZP: transitional zone pathology
BE: barium enema
PT: pull through
TA-PT: transanal pull-through
LA-PT: laparoscopic-assisted pull-through
LAR-PT: reoperation by the laparoscopic-assissted pull through
TR- PT: reoperation with the transabdominal pull through
AChE: histochemical acetylcholinesterase
HAEC: Hirschsprung-associated enterocolitis
RAIR: rectoanal inhibitory reflex
24hDAR: 24-h delayed abdominal radiograph.
